# Screening cognitive and academic problems in Duchenne Muscular Dystrophy: Validity and reliability of the Kempenhaeghe Learning Questionnaire

**DOI:** 10.1177/22143602261433149

**Published:** 2026-05-30

**Authors:** Pien MM Weerkamp, Ruben Miranda, Daniella Chieffo, Chloe Geagan, Erik H Niks, R Jeroen Vermeulen, Isabelle Desguerre, Volker Straub, Anna Wuergler Slipsager, Anna Kolesnik, Elizabeth Vroom, David Skuse, Francesco Muntoni, Eugenio Mercuri, Sylvia Klinkenberg, Jos GM Hendriksen, on behalf of the BIND Consortium Clinical Working Group

**Affiliations:** 1 3804 Kempenhaeghe Centre for Neurological Learning Disabilities, Heeze, The Netherlands; 2 School for Mental Health and Neuroscience, Maastricht University, Maastricht, The Netherlands; 3 Department of Psychobiology and Methodology in Behavioural Sciences, 16734 Universidad Complutense de Madrid, Madrid, Spain; 4 Department of Paediatric Neurology, 9371 Catholic University, Rome, Italy; 5 5994 Newcastle University, Newcastle, UK; 6 Department of Neurology, 4501 Leiden University Medical Centre, Leiden, The Netherlands; 7 Department of Neurology, 199236 Maastricht University Medical Centre, Maastricht, The Netherlands; 8 Imagine Institute des maladies genetiques Necker Enfant maladies foundation, Paris, France; 9 Department of Neurology, 5994 Danish Neuromuscular Centre, Rigshospitalet, Copenhagen, Denmark; 10 204275 Dubowitz Neuromuscular Centre, UCL Great Ormond Street, Institute of Child Health, London, UK; 11 World Duchenne Organization, Veenendaal, The Netherlands

**Keywords:** Duchenne muscular dystrophy, screening, brain related comorbidities, academics, learning difficulties

## Abstract

**Background:**

Duchenne Muscular Dystrophy (DMD) patients are at an increased risk for cognitive and academic difficulties. However, a comprehensive tool to measure DMD-specific learning and academic functioning has been lacking. To address this, we developed the Kempenhaeghe Learning Questionnaire (KLQ)

**Objective:**

This study aims to evaluate the reliability and validity of the KLQ in a large sample of individuals with DMD (*n* = 271, mean age 10, standard deviation 3) across five European countries.

**Methods:**

The KLQ is a 20-item proxy-report questionnaire based on existing instruments, designed to screen for problems in four cognitive and academic areas in DMD: Reading, Arithmetic, Memory/Attention, and Executive Functioning. Data analysis involved a principal component analysis to identify the KLQ's factor structure and a confirmatory factor analysis to verify this structure. Reliability and validity were assessed to determine the psychometric quality.

**Results:**

Factor analyses revealed four distinct but correlated dimensions of cognitive functioning and academic challenges in DMD, confirming a robust four-factor model for the KLQ. The questionnaire demonstrated good psychometric quality.

**Conclusions:**

The findings suggest that the KLQ is a useful and clinically informative, brief parent-report tool that can help clinicians identify DMD-specific key cognitive and academic challenges, with a specific focus on school-related areas such as reading, arithmetic, memory, attention, and executive functioning. While this targeted scope limits its use for assessing broader aspects of cognition, it can provide valuable insights in taking care decisions, including determining whether specialised (neuro)psychological assessment is warranted.

## Introduction

Dystrophin-related muscular dystrophies, known as dystrophinopathies, encompass a range of conditions from the severe Duchenne muscular dystrophy (DMD) to the milder Becker muscular dystrophy (BMD). DMD affects approximately 1 in 3500 to 1 in 9300 males, while BMD affects about 1 in 16,700 to 1 in 18,500 males (ORPHA: 98,896 and 98,895, respectively). These X-linked recessive inherited neuromuscular disorders primarily affect males with mutations in the *DMD* gene, the largest gene in the human genome containing 79 exons.^1,2^ The absence of functional dystrophin, as observed in DMD, and the presence of partially functional dystrophin, as seen in BMD, render muscle fibres prone to damage and degeneration. This leads to progressive muscle weakness and loss of function.^2^ The *DMD* gene comprises several tissue-specific promoters, with the full-length muscle isoform (Dp427m) predominantly expressed in skeletal and cardiac muscle tissues. Additionally, dystrophin is expressed in the brain, where it plays crucial roles in synaptic function, neuronal signalling, and neurodevelopment. High expression levels of dystrophin isoforms Dp427c, Dp140, and Dp71 have been identified in brain regions, including the amygdala, hippocampus, cerebral cortex and cerebellum (Dp427c, Dp140 and Dp71; for reviews see^3,4^). Although precise mechanisms are not fully understood, several studies suggest that the location of the mutation may play a role in the manifestation and possibly severity of neurodevelopmental problems. These studies have reveal that patients with more distal mutations, which cumulatively affect Dp140 and Dp71, have an increased risk of cognitive deficits and lower IQ.^5–14^

Regardless of mutation site, individuals with DMD have an increased risk of cognitive deficits, and lower IQ, and neuropsychiatric problems compared to the general population.^5–14^ On average, individuals with DMD exhibit IQ scores that are one standard deviation below the population mean (full scale IQ = 84).^5^ The cognitive profile in DMD is relatively well-recognized and is characterised by both strengths and weaknesses. Perceptual organisation, abstract reasoning, and visuospatial processing may be relatively well-preserved.^15–17^ In contrast, working memory,^7,9,10,18–21^ verbal/short term memory,^8,15,16,19,21–24^ attention,^8,15,16,20,25^ executive functioning,^7,19–21,26^ and automatization of academics (reading and arithmetic)^9,14,15,17,22,24,27^ are frequently affected. Furthermore, several studies indicate that these cognitive deficits can occur regardless of intellectual disability.^18,19,28^ These cognitive challenges may often interact with behavioural and socio-emotional issues, as many individuals with DMD are also at risk for behavioural, neurodevelopmental and emotional symptoms, as shown in a recent systematic review and meta-analysis.^29^ We previously described a theoretical framework to understand the brain related comorbidities in DMD.^30^ Specifically, we proposed that there are four domains of interest encompassing ten different and potential areas of problematic functioning (The Big Ten of Duchenne): intelligence, working Memory/Attention and executive function in the cognitive domain (1), reading and arithmetic in the learning domain (2), Attention Deficit Hyperactivity Disorder (ADHD), Autism Spectrum Disorder (ASD), Obsessive Compulsive Disorder (OCD) in the psychiatric domain (3), and anxiety and depression in the emotional domain (4). In clinical practice, these ten domains may show overlap.^30^

Various instruments have been used to assess behavioural and psychological functioning in DMD,^31^ but none have specifically been developed for Duchenne-related cognitive functioning. In 2024, two questionnaires have been developed to address this gap: the BELS^32^ and the DUMAND.^33^ The latter, a 45-item checklist to screen for DMD-associated neurobehavioural difficulties, assessing five categories: cognition and learning, social responsiveness, emotion regulation, externalising behaviour, and eating and sleeping.^33^ This was a pilot study incorporating item selection, expert panel assessment for face validity, comprehensiveness, and a pilot validation study in a DMD sample of 20 boys with DMD. The second study is on the newly developed 48 item BELS questionnaire: a screening for Behavioural, Emotional, Learning and Social difficulties (12 items per domain). This tool has been administered in an initial pilot study in 34 caregivers of Duchenne patients and 11 Becker patients with an age range between 4–19 year, visiting a neuromuscular clinic in the USA.

The current tool, the Kempenhaeghe Learning Questionnaire (KLQ) was subsequently designed to assess the cognitive and academic/learning areas of the Big Ten. It is expected that these cognitive and academic processes are interrelated, as these areas may show overlap.^30,34–36^ The area of intelligence was not included in the KLQ as this should be assessed with intelligence tests.^37^ In contract to the BELS and DUMAND screening tools, the KLQ only targets cognition and learning instead of also considering other challenges. This specificity of our questionnaire is not necessarily a limitation. Instead, it reflects the shared recognition of the importance of investigating cognition and learning in a way that is accessible and practical for patients, health care professionals, and caregivers. It is promising to see that different tools are being developed with this shared goal in mind. Moving forward, the primary aim of this study is to evaluate the reliability and validity of the KLQ and its psychometric quality as a screening tool for identifying learning and academic problems in a large population of individuals with DMD from different European countries.

## Methods

### Participants

The following centres participated in this study: University College London (UCL), University of Newcastle Upon Tyne (UNEW), Kempenhaeghe Centre for Neurological Learning Disabilities (KEM), Leiden University Medical Center (LUMC), Universidad Complutense de Madrid (UCM), Imagine Institute des maladies genetiques Necker Enfant maladies foundation Paris (NEM), Universita’ Cattolica del Sacro Cuore, Rome (UCSC). These are part of a larger consortium and part of the multicentre BIND project (Brain INvolvement in Dystrophinopathies (Horizon 2020 (847826), https://bindproject.eu/).

Parents of boys and adolescents with DMD were contacted by letter, e-mail, telephone, or in person to participate in this study. Approval was obtained from the local ethics committees of all participating centres. The participating DMD families were located in various regions across the Netherlands, United Kingdom (UK), France, Spain, and Italy. For further details on participant recruitment and procedures, we refer to a workshop report describing the design of the whole BIND study.^38^

The BIND project has two parts: Part 1 involves an online battery of questionnaires, and Part 2 consists of in-person neuropsychological assessment. For this study, we focus on all DMD patients who participated in Part 1 (from Spain, the UK, the Netherlands, France and Italy), and a subset of these patients who also took part in Part 2, including DMD patients from Spain, the UK, and the Netherlands. The subset of patients in Part 2 includes only those with complete and comparable data available at the time of this publication. The complete set of participants was used for all data analysis, except for correlating the data of part 1 with data from part 2. A comprehensive analysis of all the Part 2 data will be done in a separate study and is outside the scope of this current study.

The inclusion criteria for both parts of the BIND study were boys aged 5–17 years old. All patients had a molecular diagnosis of DMD. Exclusion criteria included the absence of a molecular confirmed diagnosis of DMD, the presence of any other serious neurological comorbidity (i.e., epilepsy), planned surgical intervention within 6 months from the study, and the inability to provide consent (for parents/guardians) or assent (for the participants).

### Measures

Between January 2020 and March 2024, participants and their families completed an online set of questionnaires for the BIND study. This included the KLQ, the Psychosocial Adjustment and Role Skills Scale (PARS-III^39^), and the Kempenhaeghe History Taking Questionnaire (KHTQ) for assessing patient demographic and background information. Parents were asked to fill out the questionnaires for their children. Many participants involved in this part of the BIND project also underwent comprehensive neuropsychological and academic assessments at the clinical or research centres in their respective countries. As mentioned before, for the current study, we focus specifically on the academic testing conducted in the Dutch, Spanish, and UK samples.

### The Kempenhaeghe learning questionnaire

The KLQ is a short, 20-item questionnaire designed to assess learning issues. There are two versions: proxy and self-report. This study focuses on the proxy version. Parents were asked to rate items on a five-point Likert frequency scale: (1) “Never”; (2) “Rarely”; (3) “Sometimes”; (4) “Frequently” and (5) “Always”. Following an initial trial involving 10 parents of children not affected by a neuromuscular disorder, an additional option of “Not Applicable” was included for younger children. The items are categorised into four a priori subscales, focusing on four areas: Reading, Arithmetic, Memory/Attention, and Executive Functioning. The total score is computed by summing the scores from these items, with a maximum of 100 points, where higher scores reflect greater learning difficulties. The “Not Applicable” responses were treated as missing data in the analysis. Missing responses to the items were excluded listwise to maximise the use of the available data. The psychometric properties of these measures, based on their total scores, range from acceptable to good. A native speaker proficient in neuropsychology translated items from English into Danish, Dutch, French, Italian and Spanish. To ensure the quality of the items, reverse translation (e.g., from Spanish back to English) was conducted for all languages. Although Denmark is part of the BIND consortium, no Danish participants were included in the current study sample. However, a Danish version of the KLQ was developed in anticipation of future research

The Reading and Arithmetic scales were adapted from existing items in the Colorado Learning Difficulties Questionnaire (CLDQ).^40^ We implemented the authors’ recommendation to incorporate the two suggested additional math items, which they previously tested with 70 participants to strengthen the validity of the math scale. The psychometric properties of the CLDQ Reading and Arithmetic subscales are well-established, with internal consistency coefficients of .83 and .80, respectively, and test-retest reliability coefficients of .81 for Reading and .73 for Arithmetic. To enhance clarity and comprehension for parents, three of the six items in the CLDQ Reading subscale (items 2, 3, and 5) were rephrased. The Arithmetic scale items were informed by the Woodcock-Johnson Achievement Battery,^40^ which has previously demonstrated significantly lower scores among individuals with DMD on its Arithmetic subtests.^28^ In the original American validation sample of the CLDQ, which was based on a community of five schools (n = 5031),^40^ the mean parent rating for the reading scale was 1.79 (SD = .94) and 1.73 (SD = .88) for the Arithmetic scale items. A priori cut-off scores were set at 1 SD above the validation sample mean: 2.67 for the reading scale and 2.60 for the Arithmetic scale. Following the approach of the original article, we also used composite means by calculating the total score of each subscale and dividing it by the number of items within that subscale. Items related to Memory and Executive Functioning were based on existing validated instruments: the Five to Fifteen-R (5-15R) tests,^29^ the Executive Function Behaviour Rating Inventory (BRIEF),^41^ and the Questionnaire of Memory (Q-MEM).^42^ All selected items were reviewed and refined regarding their theoretical relevance and applicability by an expert panel composed of child neurologists, psychologists, and parent representatives from the BIND consortium.

### Psychosocial adjustment and role skills scale III

This tool assessed psychosocial adjustment through a 28-item parent-completed questionnaire. Responses, using a 4-point scale, contribute to a total score and six psychosocial subscales: peer relations, dependency, hostility, productivity, anxiety/depression, and withdrawal. Higher scores indicate better adjustment. Previous research^43^ has demonstrated that the PARS-III is a valid and reliable tool for assessing psychosocial adjustment in individuals with DMD under 18 years old. The PARS-III is available in the languages of all participating countries. We included this questionnaire to measure the convergent validity of the KLQ.

### Individual psychological testing

The KLQ was validated using established psychological and cognitive testing scales collected during in-person assessments in the UK, Spain and the Netherlands.

For Language and Arithmetic performance assessment, different country-specific instruments were employed. Due to the considerable variability in how academic functioning is assessed in individuals with DMD across countries and settings,^44^ different validated instruments were used in the Netherlands, Spain, and the United Kingdom. As such, a convenience-based validation strategy was employed, reflecting real-world variability in clinical practice across settings. In the Netherlands, the Tempo Test Automatiseren (TTA; age range 6–12) and Continu Benoemen & Woorden Lezen-test (CB&WL; age range approximately 6–16 years)^45^ were utilised. In the UK, the Wechsler Individual Achievement Test, Third Edition (WIAT-III; age range 4–51), which includes Math Fluency and Word Reading subtests, was employed. In Spain, the screening tool for reading and arithmetic difficulties (PREDISCAL; age range 7–12) was used.^46^

To validate the Executive Functioning scale of the KLQ, we used the key search task from the Behavioural Assessment of Dysexecutive Syndrome for Children (BADS-C; age range 8–16).

For Memory/Attention, scores from the digit span subtest of the Wechsler Intelligence Scale for Children, Fifth Edition (WISC-V; 6–16), were used. Raw scores from all cognitive and academic tests were converted to age- and language-normed standard scores, which were subsequently converted into z-scores. A detailed description of the procedures and protocols of the BIND project can be found in our workshop report.^38^

### Procedure

Study data were collected and managed using REDCap electronic data capture tool hosted at UCL.^47,48^ Upon signing the informed consent form, participants received questionnaire links via email. In some cases, parents chose to complete the questionnaires using paper and pencil. Support was readily available via email, telephone, or in person to address any questions or concerns throughout the process.

### Statistical analysis

Data analysis was performed using SPSS (IBM, USA, version 29). Initially, demographic variables were calculated. A principal component analysis (PCA) was conducted to identify the underlying structure of the KLQ, with oblique rotation chosen due to the assumption that the underlying factors may be related. Additionally, Confirmatory Factor Analysis (CFA) was conducted using AMOS version 26 to verify the factor structure and identify the items with the highest loading on each factor.

To evaluate the goodness-of-fit for the factor solution, the Comparative Fit Index (CFI), Root mean Square Error of Approximation (RMSEA), and Standardized Root Mean Square Residual (SRMR) were calculated. According to the combination rules recommended by Hu and Bentler,^49^ a model to be deemed acceptable at least one of the following should be satisfied: CFI-value close to .95 suggest a good fit, a SRMR of ≤.08, RSMEA value ≤.06, and TLI ≥ .95. A CFI-value of .90 can be taken as acceptable, indicates a good fit. An RMSEA value between .08 and .10 indicates a fair fit.

To interpret the internal consistency, reliability coefficients (Cronbach's alpha) were calculated for the four subscales scores of the KLQ. A coefficient of α ≥ .80 was set as a minimum acceptable limit for total scores, while a coefficient of α ≥ .70 was set for the four subscales. Scores of α ≥ .80 are considered good, and scores of α ≥ .90 scores are considered excellent.^50^

Convergent validity was also assessed by correlating total scores with the PARS-III and individual cognition and academics tests. Spearman correlation coefficients were calculated because results on the KLQ were not normally distributed. The interpretation of the coefficients is as follows: 0.00–0.39 indicates a weak correlation, 0.40–0.59 indicates a moderate correlation, 0.60–0.79 indicates a strong correlation, and 0.80–1.00 indicates a very strong correlation.^51^

One-sample t-tests were conducted to compare the observed CLDQ scale means for Reading and Arithmetic with those of the normative sample (community sample).^40^

## Results

### Demographics

A total of 271 male participants were included the study (M = 10.2 years, SD = 3.3 years, range 5–17). Demographic data are shown in Table 1. As expected, the rates of previously diagnosed comorbidities by a doctor or psychologist, as reported by a parent or caregiver (i.e., not based on medical history data), were elevated in our sample.^29^

**Table 1.** Demographic data of the DMD study population in part 1 and part 2.

	Part 1 online questionnaires	Part 2 psychological assessment			
		Reading	Arithmetics	BADS-C	Digit span
Number of participants	271	58	54	55	61
Age, mean (SD) years	10.2 (3.3)	10.5 (2.3)	10.8 (2.2)	11.1 (2.1)	10.8 (2.5)
Country of Residence					
• UK	87 (32%)	43 (74%)	40 (74%)	39 (71%)	41 (67%)
• Italy	87 (32%)	-	-	-	-
• France	35 (13%)	-	-	-	-
• Spain	28 (10%)	13 (22%)	12 (22%)	14 (26%)	17 (28%)
• Netherlands	18 (7%)	2 (3%)	2 (4%)	2 (4%)	3 (5%)

Note: All participants were male. Categorical variables are presented as n (%). The United Kingdom (UK) data includes patients from both University College London (UCL) and University of Newcastle Upon Tyne (UNEW), data from the Netherlands include patients from Kempenhaeghe and Leiden University Medical Centre (LUMC). Abbreviations: BADS-C = Behavioural Assessment of the Dysexecutive Syndrome for Children.

### Factor analysis for learning and academic problems (as reported by parents)

The underlying factors of the questionnaire for parents’ ratings of learning and academic problems were examined using PCA. This analysis can result in items having significant loadings on more than one factor. The four-factor solution could be interpreted as follows: (1) Reading: 6 items; eigenvalue: 8.91, percentage explained variance 44.6%; (2) Arithmetic: 5 items; eigenvalue: 2.33, percentage explained variance 11.7%; (3) Memory/Attention: 5 items; eigenvalue: 2.1, percentage explained variance 10.5%; (4) Executive Functioning: 4 items; eigenvalue: 1.0, percentage explained variance 5.1%. Subsequently, a CFA was conducted on the 20 items. Main results are shown in Table 2 with item-factor correlations presented in italic and factor loadings > .40 in bold. Two items of the Memory/Attention subscale also loaded on the ‘Executive Functioning’ scale. The Kaiser–Meyer–Olkin (KMO) measure verified the sampling adequacy for the analysis, KMO = .91. Bartlett's test of sphericity χ2 (190) = 3113.89, *p* < .001, indicated that correlations between items were sufficiently large for PCA. The results from CFA demonstrated a fair fit, with a CFI of .89, TLI of .89, a SRMR was .06 and RSMEA of .09.

**Table 2.** Item-description and results of confirmatory factor analysis for the Kempenhaeghe Learning Questionnaire (KLQ).

Item	F1	F2	F3	F4
1. He has difficulty with spelling	**0** **.** **78**	0.18	0.25	0.80
2. He muddles letters up that are similar in shape and sound	**0**.**78**	0.12	0.13	0.23
3. He has difficulty pronouncing some words	**0**.**61**	−0.33	−0.02	0.32
4. He reads slowly	**0**.**78**	0.25	0.19	−0.13
5. His reading ability isńt as good as other children of his age	**0**.**82**	0.17	0.13	0.15
6. He is receiving extra help in school because of difficulties with his reading and/or spelling	**0**.**68**	0.25	0.04	0.12
7. His maths is worse than his reading and spelling	0.13	**0**.**83**	0.17	0.08
8. He makes silly mistakes in maths	0.10	**0**.**84**	0.20	0.11
9. He has trouble learning new rules in maths	0.17	**0**.**91**	0.12	0.17
10. He has difficulty learning the rules in maths	0.23	**0**.**85**	0.10	0.25
11. He has difficulty with math word problems	0.28	**0**.**82**	0.12	0.16
12. When given multiple instructions. it is hard for him to remember them all at the same time	0.31	0.33	0.31	**0**.**47**
13. He has difficulty remembering where he has put things	0.10	0.11	**0**.**41**	**0**.**62**
14. He has difficulty remembering rhymes by heart	0.30	0.24	0.08	**0**.**77**
15. He has difficulty retelling a story	0.18	0.18	0.09	**0**.**79**
16. He is forgetful during daily activities	0.05	0.12	**0**.**56**	**0**.**63**
17. He gets upset if plans change at the last minute	0.16	0.11	**0**.**49**	0.18
18. He has difficulty planning and organising daily activities	0.18	0.26	**0**.**62**	0.34
19. He leaves tasks until the last minute	0.00	0.23	**0**.**83**	0.07
20. He leaves a messy desk behind at school or at home	0.18	0.02	**0**.**76**	0.06

Note: Bold values indicate the highest loading factors in the factor analysis. F1, Reading; F2, Arithmetics; F3, Executive Functioning; F4, Memory/Attention.

### Reliability

Internal consistency of the 20-item parent report was excellent, with a Cronbach's alpha of .93. The internal consistency of the four separate subscales ranged from good to excellent: .91 for the Reading subscale, .94 for the Arithmetic subscale, .85 for the Memory/Attention subscale, and .80 for the Executive Functioning subscale.

### External validity analyses

Convergent validity of the KLQ was assessed by calculating Spearman's correlation between the total scores on the KLQ and the total scores of the PARS-III. The results indicate a significant moderate negative correlation (*r* = −.41, *p* < .001, n = 270), suggesting that higher and therefore better psychosocial adjustment scores are moderately related to fewer learning problems, as expected.

The Reading and Arithmetic subscales were significantly correlated with the standardised scores from the individual psychological testing for academics: Reading (*n* = 58): (*r* = −.703, *p* < .001) and Arithmetic (*n* = 54): (*r* = −.676, *p* < .001). This indicates that greater parental reported learning problems of reading and arithmetic were associated with lower academic test scores. The Memory/Attention subscale was significantly correlated with the digit span subtest of the WISC-V in the expected direction: high parental report on memory problems is negatively correlated with digit span score of WISC-V: (n = 74): (*r* = −.368, *p* = .001). The Executive Functioning did not significantly correlate with the key search task from the BADS-C (n = 59): (*r* = −.225, *p* = .086). Spearman correlations between the four factors are reported in Table 3. Patterns of subscale intercorrelations provide data for construct validity. The correlations ranged from .36 to .65 (*p* < .001), indicating significant relationships among the subscales.

**Table 3.** Correlation matrix (Spearman coefficients) for the four subscales.

	Reading	Arithmetics	Executive functioning	Memory/ Attention
Reading	1.00			
Arithmetics	.51*	1.00		
Executive Functioning	.40*	.36*	1.00	
Memory/Attention	.55*	.61*	.65*	1.00

Note: *p < .001.

### Correlation between age and learning and academic difficulties

There was a weak but negative correlation between KLQ reading scale and age (*r* = −.20, *p* < .001). None of the other subscales or the total scale showed significant correlations with age. KLQ total score (*r* = −.04, *p* = .582), Arithmetic subscale (*r* = .09, *p* = .142), Executive Functioning subscale (*r* = −.03, *p* = .658), and Memory/Attention subscale (*r* = −.07, *p* = .270).

### Comparison of reading and arithmetic scores with other reported study samples

Mean parent ratings for the Reading scale items in the DMD sample (*n* = 259; mean = 2.33, SD = 1.21) were significantly higher than the ratings reported by parents in the original community sample (*t*[258] = 7.16, *p* < 001), suggesting that the present DMD sample showed more reading problems (see Table 4). Executive Functioning and Memory/Attention were not validated against a community sample because no reference or norm data are available as these scales were specifically developed for this study. Of the current DMD sample, 43.4% scored above the Reading cut-off score relative to the community norms.

**Table 4.** Composite means of learning and academic problems as reported by parents of children with DMD.

	DMD	Community	One sample
	(*n* = 271)	(*n* = 5031)	t-test
Reading	2.33 (1.2)	1.79 (0.9)	7.16*
Arithmetics	3.41 (1.0)	1.73 (0.9)	25.96*
Executive functions	2.44 (1.1)	-	
Memory/Attention	2.15 (1.0)	-	
KLQ Total score	50.60 (17.9)	-	

Note: Composite means were compared with the validation sample of the Colorado Learning Difficulties Questionnaire when possible.^40 Values represent composite mean scores for the KLQ subscales and the KLQ total score, with standard deviations in parentheses. *p* < .001.

Mean ratings for Arithmetic subscale items were also higher in the DMD sample (*n* = 251; mean = 3.41, SD = 1.03) than in the community sample (*t*[248] = 25.96, *p* < .001). This suggests that the present DMD sample showed more impairments in arithmetic compared to the community sample. On parent-reported arithmetics problems, 65.1% of the present DMD sample scored above the cut-off relative to the community norm group.

### Psychological testing scores for reading and arithmetic

In the subset of our DMD sample that underwent individual psychological testing (reading *n* = 54, and arithmetic *n* = 54): 29.3% (*n* = 17) scored 1 SD below the mean (z ≤ –1) on the reading tests, and 44.4% (*n* = 24) on the arithmetic tests.

## Discussion

This study, involving 271 children and adolescents with DMD from five different European countries (UK, Netherlands, Spain, Italy, and France), examined the psychometric quality of the KLQ, a novel parent-report screening questionnaire for cognitive and academic problems in DMD. Four areas of the Big Ten of Duchenne^30^ were included. Exploratory and confirmatory factor analyses of the KLQ revealed four correlated but separable dimensions of cognitive functioning and academic difficulties in DMD. The factor analysis identified a robust four-factor model for the questionnaire, demonstrating strong construct validity and acceptable fit indices. Our study revealed that the theoretical model of the questionnaire encompassing two domains of the Big Ten^30^ can be assessed in a reliable and valid way in clinical practice by parental report. Although, we need to consider the reliability of parents in accurately reporting back to us, as their ability or willingness to do so may introduce bias in the data. However, given the positive correlation with the performance test, their reports seem to provide a useful and informative perspective on the child's abilities. The fact that some items showed a loading on multiple factors is consistent with the heterogeneity as reported in clinical practice. Despite items loading on multiple factors, the overall fit of the model remains robust, as evidenced by the KMO, Bartlett's test, and fit indices.

The KLQ demonstrates excellent internal consistency overall and good to excellent consistency across its subscales. It also shows moderate convergent validity with the PARS-III. This might be explained by the fact that the PARS-III includes additional dimensions not assessed by the KLQ. Therefore, the moderate correlation may indicate that the PARS-III total score reflects only a specific subset of the components contributing to the overall KLQ total score. In particular, the PARS-III subscales ‘Dependency’, ‘Withdrawal’, and ‘Productivity’ may be most relevant to the KLQ total score. Additionally, relatively strong convergent evidence was found for the Reading and Arithmetic scales, suggesting that these parent-reported subscales may be useful for screening reading and arithmetic problems in both research and clinical settings. The subscales assessing Executive Functioning, Memory/Attention, and total Learning problems also showed promising results, indicating potential utility in these domains as well, although further validation is required.

Using a cut-off score of one standard deviation above the community sample composite mean, the Arithmetic and Reading subscales (2.67 and 2.60, respectively demonstrated that scores for the present DMD sample were significantly higher on both subscales compared to the original validation sample from the Colorado study (*n* = 5031).^52^ Specifically in our study sample, 42.6% of DMD sample experiences reading problems, and 65.0% experiences arithmetic problems, based on the community norm. This is consistent with the current literature indicating that children with DMD often exhibit lower reading and arithmetic skills compared to normative data.^9,14,15,17,22,24^ This also shows that arithmetic may be a more problematic area of functioning for DMD than reading, which aligns with our preliminary results of the subset of DMD patients that underwent individual psychological assessment. In our DMD sample, 29.3% scored one standard deviation below the mean on the reading tests, while 44.4% on the arithmetic tests. To our knowledge, this is the first study to report the prevalence of arithmetic problems in boys with DMD. The current findings highlight the need to include academic skills, such as reading and mathematics, in cognitive assessments of boys with DMD, which may have important implications for educational support.

While there was a slight reduction in reading difficulties as children grow older, as indicated by the weak negative correlation on the KLQ reading scale, age did not significantly influence difficulties in overall Learning, Arithmetic, Executive Functioning, or Memory/Attention. In other words, older children do not significantly differ from younger children in terms of difficulties with overall Learning, Arithmetic, Executive Functioning, or Memory/Attention based on the parent reports. This shows that the reported learning difficulties remain relatively consistent over age, suggesting that age -as expected - is not a significant factor in these areas according to parental reports. These results align with expectations based on the existing literature, indicating that learning difficulties remain quite stable over age in DMD.^11,53^ Moreover, the higher prevalence of reading difficulties in younger children, is in accordance with the frequently reported delay in language skills observed in individuals with DMD.^11,53,54^ However, caution is warranted in interpreting these findings as for the cross-sectional design of this study and the fact that it relies on parental reports. With this design we cannot account for potential changes in cognitive difficulties over time, as our data capture only a single point in each participant's development.

Consequently, further longitudinal studies are needed to confirm these results in order to better understand the potential trajectory of cognitive and academic difficulties in DMD. The KLQ can be used as a useful instrument for further longitudinal analyses.

There are several limitations to this study. First, the construct validity for the Memory/Attention subscale, and the Executive Function subscale was weak, as we did not find significant correlations. Whereas the Key Search task from BADS-C aims to measure planning, self-organisation and monitoring skills,^55^ the items on Executive Function subscale of the KLQ may be also linked to other aspects of the executive functioning such as switching and cognitive flexibility. It is well known that executive functions consist of different components according to the DSM-5.^36^ The KLQ questionnaire and the performance-based tasks assess different aspects of executive functioning and memory (e.g., everyday behavioural regulation vs. planning) and therefore may have negatively influenced construct validity.

Secondly, parent-reported questionnaires are valuable for collecting initial insights and should ideally be complemented by performance-based testing to enhance reliability and validity of questionnaire results.^56^ Future research should provide additional validation data for this. Another limitation is the lack of normative data for the KLQ total Learning problems score as well as the Memory/Attention and Executive Functioning. This makes it difficult to determine whether a score on the KLQ is atypical or within the normal range, thus affecting the ability to determine whether a specific performance level is clinical relevant. Future research should aim to address these limitations. Collecting normative data for the KLQ Total scale, and for the Memory/Attention, and Executive Functioning would enhance the interpretability and utility of the KLQ. Moreover, while the KLQ provides valuable insights into academic functioning, its focus is largely confined to school-related areas (reading, arithmetic, attention, memory and executive function). This may be considered a limitation, especially in contrast to broader tools like BELS or DUMAND. However, the KLQ was intentionally constructed as a brief screening tool to identify children with DMD at higher risk for academic and cognitive difficulties.

In conclusion, the results of our study suggest that the KLQ is a useful, brief parent report tool with good psychometric properties for clinicians to screen for DMD specific cognitive and academic difficulties – specifically the four areas of the Big Ten of Duchenne (i.e., Reading, Arithmetic, Executive Functioning, and Memory/Attention) – across different countries and languages. Parental reports on the KLQ can help identify children that have additional care needs. With this tool, we aim to help parents to address their children's possible learning problems. For clinicians, the KLQ provides valuable insights in taking care decisions, especially to determine whether specialised (neuro)psychological assessment is warranted. Given the predictive values of the math and reading subscales, the administration of the KLQ may be a cost-effective and time-efficient approach to determine when further academic evaluation is necessary.
